# Supplementary material for: Modeling glioblastoma heterogeneity as a dynamic network of cell states
Source: Mol Syst Biol. 2021 Sep 16;17(9):e10105. doi: 10.15252/msb.202010105 (PMC8444284; doi:10.15252/msb.202010105)
Supplement: Supplementary file 5 — Source Data for Figure 3 [file MSB-17-e10105-s001.zip › Figure3A_sourcedata/GSEA_3065/hallmarks_state4.GseaPreranked.1623416296068/HALLMARK_INFLAMMATORY_RESPONSE.html]

Details for gene set HALLMARK\_INFLAMMATORY\_RESPONSE[GSEA]

|  || Dataset | state4 |
| Phenotype | NoPhenotypeAvailable |
| Upregulated in class | na\_neg |
| GeneSet | HALLMARK\_INFLAMMATORY\_RESPONSE |
| Enrichment Score (ES) | -0.4029552 |
| Normalized Enrichment Score (NES) | -1.3603154 |
| Nominal p-value | 0.05293276 |
| FDR q-value | 0.14864103 |
| FWER p-Value | 0.9 |
Table: GSEA Results Summary

  

Fig 1: Enrichment plot: HALLMARK\_INFLAMMATORY\_RESPONSE      
 Profile of the Running ES Score & Positions of GeneSet Members on the Rank Ordered List

  

| PROBE | GENE SYMBOL | GENE\_TITLE | RANK IN GENE LIST | RANK METRIC SCORE | RUNNING ES | CORE ENRICHMENT || 1 | CDKN1A |  |  | 319 | 0.171 | -0.0196 | No |
| 2 | KIF1B |  |  | 437 | 0.145 | -0.0115 | No |
| 3 | SRI |  |  | 458 | 0.143 | 0.0119 | No |
| 4 | BTG2 |  |  | 689 | 0.116 | -0.0037 | No |
| 5 | CALCRL |  |  | 900 | 0.096 | -0.0197 | No |
| 6 | BST2 |  |  | 953 | 0.092 | -0.0111 | No |
| 7 | NDP |  |  | 968 | 0.091 | 0.0035 | No |
| 8 | AHR |  |  | 1147 | 0.079 | -0.0105 | No |
| 9 | SLC4A4 |  |  | 1160 | 0.078 | 0.0021 | No |
| 10 | ABCA1 |  |  | 1654 | 0.057 | -0.0668 | No |
| 11 | RELA |  |  | 1693 | 0.055 | -0.0626 | No |
| 12 | ITGB8 |  |  | 1765 | 0.053 | -0.0641 | No |
| 13 | ABI1 |  |  | 1956 | 0.048 | -0.0858 | No |
| 14 | CSF1 |  |  | 2180 | 0.042 | -0.1140 | No |
| 15 | RHOG |  |  | 2196 | 0.042 | -0.1086 | No |
| 16 | IRF7 |  |  | 2713 | 0.030 | -0.1862 | No |
| 17 | ACVR2A |  |  | 2753 | 0.030 | -0.1870 | No |
| 18 | RIPK2 |  |  | 2812 | 0.028 | -0.1911 | No |
| 19 | P2RX7 |  |  | 2919 | 0.026 | -0.2033 | No |
| 20 | NAMPT |  |  | 3040 | 0.024 | -0.2181 | No |
| 21 | F3 |  |  | 3133 | 0.022 | -0.2288 | No |
| 22 | IFNGR2 |  |  | 3147 | 0.022 | -0.2268 | No |
| 23 | KLF6 |  |  | 3920 | -0.005 | -0.3502 | No |
| 24 | PDE4B |  |  | 4063 | -0.013 | -0.3707 | No |
| 25 | TAPBP |  |  | 4109 | -0.016 | -0.3750 | No |
| 26 | PSEN1 |  |  | 4117 | -0.017 | -0.3730 | No |
| 27 | GABBR1 |  |  | 4170 | -0.020 | -0.3777 | No |
| 28 | OSMR |  |  | 4176 | -0.020 | -0.3747 | No |
| 29 | CX3CL1 |  |  | 4209 | -0.023 | -0.3757 | No |
| 30 | PDPN |  |  | 4220 | -0.023 | -0.3730 | No |
| 31 | EIF2AK2 |  |  | 4256 | -0.025 | -0.3739 | No |
| 32 | ATP2A2 |  |  | 4371 | -0.034 | -0.3860 | No |
| 33 | GNAI3 |  |  | 4426 | -0.037 | -0.3877 | No |
| 34 | HAS2 |  |  | 4502 | -0.045 | -0.3915 | Yes |
| 35 | SLC11A2 |  |  | 4513 | -0.045 | -0.3848 | Yes |
| 36 | ADORA2B |  |  | 4561 | -0.049 | -0.3833 | Yes |
| 37 | IFNAR1 |  |  | 4576 | -0.049 | -0.3764 | Yes |
| 38 | MYC |  |  | 4618 | -0.053 | -0.3732 | Yes |
| 39 | ADRM1 |  |  | 4687 | -0.059 | -0.3732 | Yes |
| 40 | RAF1 |  |  | 4821 | -0.069 | -0.3818 | Yes |
| 41 | SLC7A1 |  |  | 4953 | -0.079 | -0.3882 | Yes |
| 42 | TPBG |  |  | 4976 | -0.081 | -0.3768 | Yes |
| 43 | TNFRSF1B |  |  | 5081 | -0.090 | -0.3769 | Yes |
| 44 | IRF1 |  |  | 5157 | -0.098 | -0.3708 | Yes |
| 45 | PLAUR |  |  | 5171 | -0.099 | -0.3545 | Yes |
| 46 | LY6E |  |  | 5230 | -0.104 | -0.3446 | Yes |
| 47 | PTPRE |  |  | 5306 | -0.110 | -0.3363 | Yes |
| 48 | ATP2B1 |  |  | 5594 | -0.144 | -0.3558 | Yes |
| 49 | CD82 |  |  | 5621 | -0.147 | -0.3327 | Yes |
| 50 | KCNJ2 |  |  | 5756 | -0.174 | -0.3220 | Yes |
| 51 | SERPINE1 |  |  | 5812 | -0.186 | -0.2963 | Yes |
| 52 | MET |  |  | 5815 | -0.187 | -0.2619 | Yes |
| 53 | AXL |  |  | 5826 | -0.189 | -0.2283 | Yes |
| 54 | LDLR |  |  | 5843 | -0.194 | -0.1949 | Yes |
| 55 | TNFAIP6 |  |  | 5872 | -0.199 | -0.1624 | Yes |
| 56 | SELENOS |  |  | 5922 | -0.213 | -0.1308 | Yes |
| 57 | EMP3 |  |  | 6085 | -0.287 | -0.1035 | Yes |
| 58 | DCBLD2 |  |  | 6104 | -0.300 | -0.0506 | Yes |
| 59 | TIMP1 |  |  | 6191 | -0.409 | 0.0116 | Yes |
Table: GSEA details [plain text format]

  

Fig 2: HALLMARK\_INFLAMMATORY\_RESPONSE: Random ES distribution      
 Gene set null distribution of ES for **HALLMARK\_INFLAMMATORY\_RESPONSE**
